# Divergent Associations of the VEGF (*–2578C/A*) Polymorphism with Imaging-Based Severity and Symptom Profile of Adenomyosis in Infertile Women: An Exploratory Analysis

**DOI:** 10.3390/medicina62030571

**Published:** 2026-03-19

**Authors:** Mihai Surcel, Mihaela Iancu, Ioana Cristina Rotar, Iulian Goidescu, Adelina Staicu, Georgiana Nemeti, Dan Boitor-Borza, Roxana Liana Lucaciu, Adriana Corina Hangan, Daniel Mureșan, Lucia Maria Procopciuc

**Affiliations:** 1 Department of Obstetrics and Gynecology, “Iuliu Hațieganu” University of Medicine and Pharmacy, 400015 Cluj-Napoca, Romania; mihai.surcel@umfcluj.ro (M.S.); cristina.rotar@umfcluj.ro (I.C.R.); goidescu.iulian@elearn.umfcluj.ro (I.G.); adelina.staicu@umfcluj.ro (A.S.); georgiana.nemeti@elearn.umfcluj.ro (G.N.); dan.boitor@elearn.umfcluj.ro (D.B.-B.); muresandaniel01@elearn.umfcluj.ro (D.M.); 2 Biostatistics and Medical Informatics, Faculty of Nursing and Health Sciences, “Iuliu Hațieganu” University of Medicine and Pharmacy, 400349 Cluj-Napoca, Romania; 3 Department of Pharmaceutical Biochemistry and Clinical Laboratory, Faculty of Pharmacy, “Iuliu Hațieganu” University of Medicine and Pharmacy, 400349 Cluj-Napoca, Romania; liana.lucaciu@umfcluj.ro; 4 Department of Inorganic Chemistry, Faculty of Pharmacy, “Iuliu Hațieganu” University of Medicine and Pharmacy, 400012 Cluj-Napoca, Romania; adriana.hangan@umfcluj.ro; 5 Medical Biochemistry, Department of Molecular Sciences, Faculty of Medicine, “Iuliu Hațieganu” University of Medicine and Pharmacy, 400349 Cluj-Napoca, Romania; lprocopciuc@umfcluj.ro

**Keywords:** adenomyosis, VEGF, polymorphism, ultrasound, dysmenorrhea, menorrhagia, infertility

## Abstract

*Background and Objectives:* Adenomyosis is increasingly being recognized as a heterogeneous uterine disorder with variable clinical expressions. Current ultrasound-based classifications do not consistently align structural severity with symptom burden. Given its role in angiogenesis, inflammation, and endometrial remodeling, vascular endothelial growth factor (VEGF) signaling may influence both morphological features and clinical manifestations. This study evaluated the association between three VEGF polymorphisms (*−2578C/A*, *−634G/C*, *−936C/T*) and adenomyosis presence, ultrasound-based severity, and symptoms in infertile women undergoing in vitro fertilization (IVF). *Materials and Methods*: In this prospective cohort study, 85 infertile women were assessed for adenomyosis using MUSA criteria and Exacoustos grading. VEGF genotyping was performed by PCR-RFLP. Clinical data included menstrual bleeding status scores and dysmenorrhea intensity. *Results*: Under a dominant model, the variant genotypes (AA+CA) of the VEGF*–2578C/A* polymorphism −2578 A showed increased odds of adenomyosis versus CC (adjusted OR = 4.00, 95% CI: 1.48–10.84; *p* = 0.0037). The A allele frequency was higher in women with adenomyosis (57.14% vs. 33%), which was consistent with increased susceptibility (OR = 2.71, 95% CI: 1.44–5.09; *p* = 0.003). The AA + CA genotypes were more frequent with higher ultrasound-based severity (*p* = 0.0029; 50% vs. 72% vs. 100% across increasing severity strata); however, among women with adenomyosis, AA + CA carriers had lower odds of clinically relevant dysmenorrhea (adjusted OR = 0.18, 95% CI: 0.06–0.55; *p* = 0.0017), which remained significant after correction for multiple testing (adjusted *p* = 0.0051). No significant associations were identified for *−936C/T* or *−634G/C* across adenomyosis presence, ultrasound-based severity, number of sonographic features, dysmenorrhea, or heavy menstrual bleeding (all *p* > 0.05). *Conclusions*: These preliminary findings suggest divergent associations of VEGF *−2578C/A* with structural severity versus symptom expression, supporting a partial dissociation between ultrasound-defined severity and clinical phenotype in adenomyosis.

## 1. Introduction

Adenomyosis is a multifactorial uterine disorder defined by the presence of ectopic endometrial glands and stroma within the myometrium, typically surrounded by hypertrophic and hyperplastic smooth muscle [1]. While its recognition has long relied on histological features, current perspectives increasingly frame adenomyosis as a polymorphic disease entity shaped by diverse pathogenic trajectories [2,3]. Emerging evidence implicates aberrant proliferation, defective epithelial repair, dysregulated angiogenesis, chronic inflammation, and immune dysfunction [4,5,6,7] as key modulators of both disease evolution and symptom expression. Building on this evolving understanding, the structural definition of adenomyosis is now clearly established through the MUSA-defined sonographic criteria (Morphological Uterus Sonographic Assessment consensus) [8]. However, mapping these features onto symptom-based subtypes has yielded inconsistent results. The most prominent classification systems—those of Kishi et al. (2012) [9], Chapron et al. (2020) [10], and Exacoustos et al. (2021) [11]—have shown limited ability to stratify adenomyosis by symptom burden, as none have consistently captured the full spectrum of disease expression across both structural and clinical domains.

Cohort-based studies have reported inconsistent correlations between imaging-defined subtypes and clinical outcomes—including pain, abnormal uterine bleeding, and infertility—thereby limiting their capacity to guide integrated therapeutic decision-making [12,13,14,15,16]. Although some reports have observed that dysmenorrhea and menorrhagia frequently coexist with impaired endometrial receptivity, suggesting a partial overlap between symptom expression and reproductive dysfunction, this relationship remains insufficiently characterized [16].

This fragmented, domain-specific application underscores a central limitation of current imaging-based frameworks: their inability to simultaneously reflect both symptom trajectory and reproductive prognosis in a unified model of severity. Within this framework, vascular endothelial growth factor (VEGF) signaling has emerged as a central regulator of endometrial angiogenesis, tissue remodeling, and inflammatory crosstalk—processes intimately involved in uterine physiology and frequently perturbed across gynecologic disorders [17,18,19]. Disturbances in this system—whether through hyperactivation [20,21,22] or insufficient signaling [23]—have been implicated in abnormal bleeding, impaired endometrial repair, and disruptions of implantation across a range of reproductive disorders. Histological studies in adenomyosis have similarly described altered vascular architecture, including increased microvessel density [6] and disorganized neovascularization [7], suggesting that angiogenic pathways may contribute to the broader tissue environment in which the disease develops and expresses itself. Genetic polymorphisms within the VEGF gene cluster have been examined in multiple reproductive and gynecologic contexts, including recurrent implantation failure, recurrent miscarriage, polycystic ovary syndrome, and endometriosis [24,25,26,27,28,29]. In contrast, studies focusing on adenomyosis remain scarce, leaving open important questions regarding whether VEGF-related genetic variation may contribute to the biological heterogeneity observed among affected patients [30,31]. These gaps highlight the need for investigative efforts aimed at clarifying how VEGF signaling and its genetic determinants relate to the complex and heterogeneous phenotypes of adenomyosis [5,6]. One of these polymorphisms is *−936 C/T* (rs3025039), located in the 3′ untranslated region (3′-UTR) of the VEGF gene. This genetic variation in the VEGF gene may affect mRNA stability and post-transcriptional regulation. The T allele has been associated with lower circulating and tissue VEGF levels compared with the C allele [32]. Reduced VEGF expression may impair angiogenesis and endometrial receptivity, which are processes essential for implantation and placentation. Thus, the VEGF *−936 C/T* may influence female fertility and pregnancy maintenance [33]. Another polymorphism is VEGF *−2578C/A*, located in the promoter region of the VEGF gene, which can influence gene transcription and VEGF expression levels and plays a molecular role in angiogenesis and vasculogenesis, influencing transcription factor binding in the VEGF promoter [34,35]. There are studies which have suggested that A allele is associated with lower VEGF expression as compared to the C allele [35]. The VEGF *−634 C/G* (rs2010963) polymorphism in the 5′ untranslated region (5′-UTR) of the VEGF gene influences mRNA stability and thus protein production. The C allele has frequently been associated with higher VEGF expression compared with the G allele [36]. The *−634 C/G* polymorphism is not used as an independent clinical biomarker, but has been mainly investigated as a genetic susceptibility factor, often in combination with other VEGF polymorphisms (*−2578 C/A*, *−1154 G/A*, *−936 C/T*) [33]. Moreover, these three genetic variations may affect endometrial vascular remodeling, implantation and placental development, and ovarian folliculogenesis [18,19,20].

To this end, the present investigation has been designed to delineate the association between specific VEGF polymorphisms (*−2578C/A*, *−936C/T*, *−634C/G*) and the susceptibility to adenomyosis, while systematically evaluating their relationship with imaging-based disease burden and clinical symptom expression, particularly dysmenorrhea and menorrhagia. Although the study population exclusively comprised infertile women and may not fully mirror the general population, this selection had been methodologically intentional. Infertility, particularly in the context of adenomyosis, is characterized by a significantly higher frequency and greater severity of uterine symptoms compared to fertile cohorts. Consequently, this population represents a clinically enriched framework that optimizes the probability of detecting biologically significant genotype–phenotype associations, thereby facilitating a rigorous and controlled assessment of the relationship between VEGF genetic variation and the clinical expression of the disease.

## 2. Materials and Methods

### 2.1. Study Design and Participant Recruitment

The project planning and clinical feasibility assessment commenced in May 2023. Following the receipt of IRB approval on 20 June 2023, patient enrollment and data collection were initiated for a prospective observational cohort study, which was conducted at the Assisted Reproduction Department of the First Obstetrics and Gynecology Clinic (Cluj-Napoca, Romania) from June 2023 to January 2024. The study was approved by the Ethics Committee of “Iuliu Hațieganu” University of Medicine and Pharmacy (protocol no. Av2-95/20 June 2023) and complied with the Declaration of Helsinki. A total of 85 infertile women undergoing IVF treatment were consecutively included after providing written informed consent for the use of clinical and genetic data. Women were excluded if they met any of the following criteria: aged over 40 years; untreated hydrosalpinx confirmed by imaging; moderate or severe endometriosis; known inherited thrombophilia or antiphospholipid syndrome; chromosomal abnormalities in either partner; a body mass index greater than 35 kg/m^2^; or major uterine abnormalities, including congenital anomalies, submucosal fibroids, fibroids larger than 5 cm, large endometrial polyps, or severe intrauterine adhesions (synechiae). These exclusion criteria were applied to minimize potential confounding factors that could independently influence symptom presentation. This analysis represented a secondary analysis derived from the same IVF cohort, addressing a different clinical question and employing a distinct analytical framework. Unlike the ovarian-centered study, which stratified patients by infertility etiology, the present investigation analyzed the cohort as a single group, with objectives centered on adenomyosis diagnosis, sonographic severity, symptom expression, and VEGF genotype–phenotype associations. For each participant, clinical symptomatology was recorded during direct interviews conducted by the attending physician in the month preceding ultrasound and IVF treatment. Dysmenorrhea was assessed using a visual analog scale (VAS, 0–10). For analytical purposes, dysmenorrhea was defined as clinically relevant when the VAS score was ≥4, corresponding to at least moderate pain, and was analyzed as a binary variable (present/absent) [37]. Heavy menstrual bleeding (HMB) was systematically assessed in all patients as a binary clinical variable (present/absent). A Pictorial Blood Loss Assessment Chart (PBAC) was implemented as a standardized descriptive tool across the cohort, with severe menorrhagia defined by a PBAC score ≥ 100. However, given the limited sample size and the exploratory nature of the study, PBAC-derived severity categories were reported descriptively only and were not incorporated into inferential statistical models [38].

### 2.2. Adenomyosis Diagnosis and Classification

Adenomyosis was diagnosed using the MUSA consensus sonographic trait definitions, documenting myometrial heterogeneity, wall asymmetry, myometrial cysts, hyperechogenic islands, fan-shaped shadowing, irregular or interrupted junctional zone, subendometrial lines/buds, and translesional Doppler vascularity. In accordance with MUSA recommendations, the presence of at least one direct sonographic sign was considered sufficient to establish the diagnosis.

In our cohort, 35 patients fulfilled the diagnostic criteria for adenomyosis, of which 19 exhibited moderate-to-severe imaging phenotypes, and all adenomyosis-positive patients (n = 35) were included in the uterine symptom–genotype study arm reported here. Imaging severity was graded following the Exacoustos et al. (2021) four-degree extent scoring system, applied per myometrial disease component and subsequently summed at the patient level [11]. Based on the summed extent score, imaging severity was classified as mild (1–3 points), moderate (4–6 points), or severe (≥7 points).

### 2.3. Genetic Analysis

Genotyping of VEGF polymorphisms (*−936C/T*, *−634C/G*, *−2578C/A*) was performed using PCR-RFLP methods, following the protocols of Papazoglou et al. (2004) and Liu et al. (2009) [32,39]. DNA was extracted from 5 mL EDTA whole-blood samples using a Zymo Quick-DNA Miniprep kit (Quick-DNAMiniprep, Kit-Zymo Research Corporation, Freiburg, Germany) and stored at −20 °C. PCR amplification was performed in an iCycler C1000 BioRad (Bio-Rad Life Science, Hercules, CA, USA), with amplified fragments having 208 bp (*−936C/T*), 304 bp (*−634C/G*), and 324 bp (*−2578C/A*). Specific primers (Kaneka Eurogentec S.A. Biologics Division, Liege, Belgium) were used for each polymorphism for each genetic variations and standard PCR reagents. RFLP analysis was conducted with specific restriction enzymes (New England Biolabs (New England Biolabs UK, Ltd., Hitchin, UK)): *NlaIII* (VEGF *−936C/T*), *BsmFI* (VEGF *−634C/G*), and *BgIII* (VEGF *−2578C/*A). The amplified and digested fragments were resolved on 3% agarose gels. The genotypes were determined based on fragment sizes: VEGF *−936C/T* (C allele = 208 bp; T allele = 122, 86 bp; CC = 208 bp; CT = 208, 122, 86 bp; TT = 122, 86 bp); VEGF *−634C/G* (C allele = 304 bp; G allele = 193, 111 bp; CC = 304 bp; CG = 304, 193, 111 bp; GG = 193, 111 bp); and VEGF *−2578C/A* (C allele = 324 bp; A allele = 202, 122 bp; CC = 324 bp; CA = 324, 202, 122 bp; AA= 202, 122 bp). Details of the methods used were presented by Procopciuc et al. (2025) [33].

### 2.4. Statistical Analysis

Quantitative characteristics were summarized using arithmetic mean and standard deviation for variables which followed a normal distribution or median with an interquartile range (IQR: [Q1, Q3] where Q1 is first quartile and Q3 is third quartile) for variables that were not normally distributed. Normality was assessed by Shapiro–Wilk tests, visual inspections of quantile–quantile plots (Q-Q plots), and descriptive measures of the shape of empirical distribution (univariate skewness and kurtosis). Comparative analyses of allele and genotype frequencies of VEGF gene polymorphisms between infertile women with adenomyosis and the non-adenomyosis groups were tested using Chi-square or exact Fisher’s test. The departure from Hardy–Weinberg equilibrium (HWE) for the studied SNPs was tested using the exact Chi-square test from “SNPassoc” R package [40]. The association between the studied VEGF gene polymorphisms and the odds of adenomyosis, dysmenorrhea or menorrhagia was evaluated by unconditional binomial logistic regression testing under codominant, dominant, and recessive genetic models. To control the family-wise error rate, False Discovery Rate (FDR)-corrected *p*-values were reported. The effect size of association was estimated using the adjusted odds ratio (OR) with 95% confidence interval (95% CI). Comparative analysis of haplotype frequencies between the studied groups was based on haplotype-based generalized linear models (GLM) using the R-project package “haplo.stats” [41]. All statistical analysis was performed in R software, version 4.5.1 [42]. Statistical significance as defined as *p* < 0.05 for all inferential methods.

## 3. Results

The characteristics of the infertile women included in the current study are presented in Table 1. The mean age of the infertile women ranged from 22 to 39 years, with a mean of 34.29 (3.87) years. Regarding infertility diagnosis, idiopathic infertility was the most frequent type, followed by tubal factors (23.53%), while infertile women with polycystic ovary syndrome accounted for 20% of all infertility cases. The median duration of infertility was 4 years (IQR: 3–6), with 60% having an infertility duration longer than 3 years. Adenomyosis was diagnosed in 35 of 85 patients (41.2%) (Table 1).

Among the 35 infertile women diagnosed with adenomyosis, clinically relevant dysmenorrhea (VAS ≥ 4) was observed in 57.1% of patients, while severe menorrhagia was present in 14.28%. The median adenomyosis severity score was 4.0 (IQR: 2.0–7.0). Diffuse adenomyosis was the most common subtype (40%), followed by focal (31.4%), mixed-type (20%), and adenomyoma (8.6%). Regarding individual MUSA features, echogenic subendometrial lines or buds were the most frequent finding (68.5%), followed by irregular or interrupted junctional zone (57.1%) and hyperechogenic islands (54.2%). Myometrial cysts and asymmetric wall thickening were each present in 51.4% of cases, while fan-shaped shadowing and translesional vascularity were observed in 40% of patients (Table 2).

### 3.1. Association Between the VEGF –936C/T, VEGF –634C/G, and VEGF –2578C/A Gene Polymorphisms and the Odds of Adenomyosis

The distributions of the VEGF *–2578C/A*, *–936C/T*, and *–634C/G* did not significantly deviate from those expected under Hardy–Weinberg equilibrium in infertile women with and without adenomyosis (p_HWE_ > 0.05). The genotype distributions of the VEGF *–2578C/A* polymorphism was significantly different in the infertile women with adenomyosis and those without adenomyosis (Table 3). The variant genotype (A/A + C/A) was significantly associated with increased odds of adenomyosis compared to the C/C genotype under the dominant model (OR = 4, 95% CI: [1.48, 10.84]. The association remained statistically significant after adjusting for age (adjusted *p*-value = 0.0037). In the infertile women with adenomyosis, the frequency of the A allele of VEGF *–2578 C/A* gene polymorphism was higher than that in infertile women without adenomyosis (57.14% vs. 33%), and the presence of A allele was significantly associated with an increased susceptibility to adenomyosis (OR = 2.71, 95% CI: [1.44, 5.09], *p* = 0.003).

### 3.2. Association of Haplotypes of VEGF Gene Polymorphisms with the Odds of Adenomyosis

The VEGF gene polymorphisms displayed a weak pairwise linkage disequilibrium (D’_VEGF -_*_936_*_/-_*_634_* = 0.29, *p* = 0.00039, D’_VEGF -_*_634_*_/-_*_2578_* = 0.02, *p* = 0.9138, D’_VEGF_ *_-936/-2578_* = 0.09, *p* = 0.4645) in all samples of the infertile women. The C-C-C was the most common three-locus haplotype in all samples of the infertile women (38.41%). Compared with the haplotype of C-C-C, the C-C-A haplotype exhibited a significant increase in the odds of developing adenomyosis after adjusting for patients’ age (Table 4). Concerning two-locus haplotypes models, the regression analysis highlighted that VEGF –*634*/–*2578* C-A (adjusted OR = 2.08, 95% CI: [1.04, 4.16]) and VEGF –*936*/–*2578* C-A (adjusted OR = 2.86, 95% CI: [1.31, 6.26]) haplotypes were also significant risk factors for adenomyosis development after adjusting for patient’s age.

### 3.3. Association Between the VEGF –936C/T, VEGF –634C/G, and VEGF –2578C/A Gene Polymorphisms and the Severity of Adenomyosis

The genotype frequencies of the VEGF *–936C/T* and VEGF *–634C/G* gene polymorphisms did not significantly differ according to adenomyosis severity in the codominant model (Table 5). Under the dominant model of the VEGF *–2578C/A* polymorphism, a significant difference (*p* = 0.0029) in frequency of the variant genotype (AA + CA) was observed across adenomyosis severity groups, with patients with severe adenomyosis showing a higher frequency of the variant genotype (100%) compared to those with moderate adenomyosis (75%) or without adenomyosis (50%).

### 3.4. Association Between the VEGF –936C/T, VEGF –634C/G, and VEGF –2578C/A Gene Polymorphisms and the Odds of Clinically Relevant Dysmenorrhea and Severe Menorrhagia

Under the dominant model, carriers of the variant genotype (AA + CA) of VEGF *–2578C/A* polymorphism had significantly lower odds of clinically relevant dysmenorrhea compared to the CC genotype (Table 6). The association remained significant after adjusting for age (adjusted *p* = 0.0017, adjusted OR = 0.18, 95% CI: 0.06–0.55) and remained robust following correction for multiple testing (pFDR = 0.0051). Although the variant genotype (AA + CA) was also associated with lower odds of severe menorrhagia (adjusted *p* = 0.0230, adjusted OR = 0.20, 95% CI: 0.05–0.86), the association did not remain statistically significant after correction for multiple testing (Table 7).

## 4. Discussion

In this cohort, under the dominant model, A allele carriers of the VEGF *−2578C/A* polymorphism (CA + AA) were significantly associated with the presence of adenomyosis, representing the only variant among those examined to show a clear relationship with disease occurrence. Consistently, haplotype-based analysis identified significant associations for the three-locus VEGF haplotype −936C/−634C/−2578A, as well as for two-locus haplotypes combining −634C/−2578A and −936C/−2578A, all of which were linked to increased odds of adenomyosis compared with reference configurations. When stratifying by imaging-defined severity, the frequency of the CA/AA genotypes of the VEGF *−2578C/A* polymorphism increased progressively from women without adenomyosis to those with moderate and, most prominently, severe forms, indicating a graded association along the morphological spectrum. However, in this dataset, imaging-defined severity did not consistently parallel clinical presentations, and these dimensions should therefore be considered separately when evaluating genotype–phenotype relationships. Consistent with this distinction, the VEGF *−936C/T* and *VEGF−634C/G* polymorphisms did not differ across imaging severity groups. Regarding symptom severity, the VEGF *−2578C/A* polymorphism (CA + AA) variant exhibited markedly lower odds of dysmenorrhea under the dominant model, whereas a similar trend for severe menorrhagia did not remain significant after correction for multiple testing. The principal finding of this study was the strong association between the VEGF *−2578C/A* polymorphism and adenomyosis, with the CA/AA genotypes correlating with both disease presence and a higher number of sonographic features, translating into greater severity according to current imaging-based classification systems. These findings have not negated the relevance of imaging-defined disease burden; rather, they could suggest that morphological extent and symptom severity may follow partially independent biological trajectories. The study cohort comprised older, long-term infertile women with a substantial symptom burden. This clinical profile likely enriched the sample for adenomyosis and extensive sonographic involvement, thereby increasing the power to detect genotype associations. In this context, the high prevalence of adenomyosis and lesion load reflected the characteristics of the selected population and served to contextualize rather than drive the observed genotype–phenotype relationships. Although the mechanistic basis of the VEGF *−2578C/A* polymorphism (CA + AA) association has not been addressed directly in this study, several biologically plausible pathways have been described in the literature. VEGF variants associated with lower transcriptional activity have been linked to impaired angiogenesis and chronic junctional zone hypoxia [43], defective basal endometrial repair within the TIAR framework, fibro-inflammatory remodeling mediated by TGF-β and MMP activation, and sterile inflammation driven by hypoxic signaling [44]. These processes collectively outline a coherent biological context for how VEGF modulation might influence adenomyosis-related tissue remodeling. Equally noteworthy was the distinct pattern observed for clinical symptomatology. The *VEGF* −2578CC genotype—typically associated with higher VEGF expression—was linked to significantly increased odds of severe dysmenorrhea and showed a trend toward more frequent menorrhagia. This observation aligned with existing evidence implicating VEGF in enhanced neurogenesis and amplified nociceptive signaling [45]. Additionally, hypoxia-induced stabilization of HIF-1α can activate NF-κB-dependent transcription of VEGF and multiple inflammatory mediators [46,47,48,49]. Increased angiogenesis and the presence of fragile, highly permeable microvessels have also been linked to abnormal uterine bleeding in adenomyosis [50,51,52]. What distinguishes our findings from the prevailing understanding of adenomyosis was not simply the non-parallel pattern implied by the genotype–imaging and genotype–symptom associations—which has been noted in other cohorts—but the direction of this divergence within our dataset. While VEGF *−2578C/A* polymorphism (CA + AA) was associated with the presence of adenomyosis and a higher number of ultrasound features, the CC genotype—which is linked to higher VEGF expression—clustered among women with the most intense symptoms. This pattern ran counter to the commonly assumed model in which an increasing lesion load translates into more severe clinical manifestations [14,53]. To account for this apparent inversion, a speculative but biologically plausible explanation may be considered. Imaging-defined “severity” can reflect the cumulative impact of molecular remodeling processes, such as deep myometrial infiltration [2,3,54], junctional-zone disruption [3], and progressive fibrogenesis [3,6]—the latter driven by TGF-β-mediated fibroblast activation [5,55], extracellular matrix deposition [5,56] and myofibroblast differentiation [5,54,56]. In contrast, symptom generation appeared more tightly linked to pathways such as angiogenesis [6,43,46], neurogenesis [57,58], inflammatory signaling [6,55,56,57], vascular permeability [6,44], and discrete components of the fibrogenic program that interface with nociceptive and hemodynamic regulation [54,59]. If these domains are only partially coupled, as emerging evidence would suggest, then genetic variants influencing VEGF signaling could shape structural and symptomatic phenotypes along different trajectories. Under this framework, VEGF *−2578C/A* polymorphism (CA + AA) variants may favor a phenotype characterized by impaired angiogenesis, defective repair, and enhanced fibro-inflammatory remodeling, whereas the *CC* genotype, via higher VEGF activity, may amplify neurogenic and angiogenic pathways that potentiate nociception and abnormal bleeding. This interpretation does not claim mechanistic proof but can provide a coherent context for understanding why morphological severity and symptom intensity may diverge, and how VEGF-related genetic variation could differentially influence these two dimensions of disease expression. Current research agendas have increasingly prioritized the elucidation of adenomyosis pathogenesis and the development of biologically informed classification frameworks, with growing interest in genetic and molecular contributors [5,6,7]. Although VEGF *−2578C/A* polymorphisms have only been sparsely investigated in adenomyosis or endometriosis [30,60] and more frequently examined in neoplastic contexts [61,62,63,64,65] with heterogeneous results, the prevailing hypothesis in the gynecologic literature, particularly among Asian cohorts, is that transcriptionally active CA/CC genotypes promote excessive angiogenesis and thereby increase susceptibility to adenomyosis [30,65]. This view is reinforced by multiple independent lines of evidence: increased VEGF expression in eutopic and ectopic endometrium [66,67], elevated microvessel density [20,21], enhanced endothelial proliferation, and consistent upregulation of angiogenic mediators in transcriptomic and immunohistochemical studies [20,21,43,68]. Collectively, these findings have strengthened the widely held assumption that heightened angiogenic drive is central to the initiation of adenomyotic lesions. At the same time, several oncologic and tissue repair studies have suggested that VEGF variants AA/AC associated with lower transcriptional activity may compromise stromal integrity and impair regenerative capacity, potentially facilitating tissue infiltration through mechanisms not strictly dependent on angiogenic excess [61,62]. Within this evolving landscape, the transcriptomic analysis by Juárez-Barber et al. reported a reduction in VEGF ligand–receptor interactions in the eutopic endometrium of women with adenomyosis [23]—an observation that broadened the conceptual field beyond a uniform hyperangiogenic model and raised the possibility of divergence in VEGF signaling across biological compartments. Against this backdrop, our observation that VEGF *−2578C/A* polymorphism (CA + AA) clustered among women with more pronounced morphological involvement—despite most genetic studies identifying the CC genotype as the principal susceptibility allele [60,65,66,69]—does not contradict the broader scientific trajectory. Rather, it can point to the possibility that VEGF-related pathways may exert distinct effects across biological compartments and pathological processes, with molecular signals governing tissue infiltration, fibrogenesis, angiogenesis, and nociception not necessarily moving in parallel. Under this framework, hypoproductive variants may influence structural remodeling at the endometrial–myometrial interface, whereas transcriptionally active variants may preferentially amplify the neuroangiogenic and inflammatory circuits that drive pain and abnormal bleeding. Such a process-dependent divergence would be consistent with emerging transcriptomic data [23] and may help reconcile why morphological severity and symptom burden can be mapped to different VEGF-linked biological axes.

Traditionally, small-cohort polymorphism studies have been considered hypothesis-generating and should be interpreted with appropriate methodological caution. The present investigation has been positioned within this exploratory framework. Within the evolving molecular landscape, single-gene findings may acquire contextual relevance when integrated into multidimensional models of disease biology. When considered alongside transcriptomic signatures related to endometrial receptivity, stromal remodeling, or inflammatory activation—and potentially analyzed through integrative modeling approaches—such data may contribute to the development of more refined molecular stratification frameworks. Importantly, these integrative perspectives do not increase the intrinsic statistical strength of individual variants, but they may help contextualize preliminary associations within biologically coherent pathways. In this context, the identification of VEGF-related genetic variation may, if independently validated, contribute to improved risk profiling or earlier biological characterization of adenomyosis subtypes in the future. Furthermore, emerging organoid-based and physiomimetic systems may eventually allow functional validation of mechanistically defined subgroups, including those characterized by altered angiogenic signaling, and could provide experimental platforms for testing targeted interventions, such as anti-angiogenic strategies [70,71]. At present, however, such translational implications remain conceptual and require substantial confirmatory evidence. Several limitations must be acknowledged. The cohort size could constrain the precision and stability of effect estimates. The study population represents a defined clinical subset—infertile women with a high symptom burden—which could limit generalizability. VEGF expression and protein levels were not directly measured; therefore, mechanistic inferences remain indirect. Residual confounding factors cannot be fully excluded. When taken together, the findings should be interpreted proportionately and regarded as preliminary signals within a defined clinical context, requiring validation in larger, prospectively designed studies incorporating molecular and functional assessment.

## 5. Conclusions

In summary, our findings suggest that VEGF-related genetic variation—particularly the VEGF *−2578C/A* polymorphism—may contribute to the biological heterogeneity of adenomyosis by influencing structural and symptomatic dimensions through partly distinct pathways. Although the results are exploratory, they complement emerging molecular data and could inform future efforts to develop molecularly informed classification frameworks. Further validation in larger, multi-omic cohorts is essential to determine the broader clinical applicability of these findings.

## Figures and Tables

**Table 1:** Demographic and clinical characteristics of infertile women.

Variables	Infertile Women (*n* = 85)
Age, (years) ^(1)^	34.29 (3.87)
Body mass index (BMI), (kg/m^2^) ^(1)^	23.28 (1.60)
Infertility diagnosis, *n* (%)	
Male sex, *n* (%)	17 (20.0)
Idiopathic, *n* (%)	31 (36.47)
Tubal factor, *n* (%)	20 (23.53)
Polycystic Ovary Syndrome, *n* (%)	17 (20.00)
Duration of infertility (years) ^(2)^	4 [3, 6]
Duration of infertility > 3 years, *n* (%)	51 (60.00)
Thickness of endometrium (mm) ^(1)^	9.15 (2.02)
Endometrium volume (mL) ^(2)^	4.10 [3.10, 5.20]
Adenomyosis, *n* (%)	35 (41.18)
Severe menorrhagia, *n* (%)	10 (11.76)
Clinically relevant dysmenorrhea, *n* (%)	34 (40.00)
Uterine leiomyoma, *n* (%)	23 (27.06)

Data presented as ^(1)^ arithmetic mean (sample standard deviation) or ^(2)^ median, IQR: [Q1, Q3] where Q1: first quartile; Q3: third quartile; or *n* = number of cases.

**Table 2:** Descriptive clinical and sonographic characteristics of patients with adenomyosis.

Variables	Infertile Women with Adenomyosis (*n* = 35)
Severe menorrhagia, *n* (%)	5 (14.28)
Clinically relevant dysmenorrhea (VAS ≥ 4), *n* (%)	20 (57.1)
Adenomyosis severity score, median [IQR]	4.0 [2.0–7.0]
Adenomyosis type, *n* (%)	
Focal	11 (31.4)
Diffuse	14 (40)
Adenomyoma	3 (8.6)
Mixed-type	7 (20)
MUSA sonographic features, *n* (%)	
Myometrial cysts	18 (51.4)
Hyperechogenic islands	19 (54.2)
Echogenic sub endometrial lines/buds	24 (68.5)
Fan-shaped shadowing	14 (40)
Irregular/interrupted junctional zone	20 (57.1)
Asymmetric wall thickening	18 (51.4)
Translesional vascularity	14 (40)

Data presented as median, IQR: [Q1, Q3] where Q1: first quartile; Q3: third quartile; or *n* = number of cases.

**Table 3:** Distributions of genotype frequencies of studied polymorphisms between the studied groups.

Genetic Models	Infertile Women without Adenomyosis (n_1_ = 50)	Infertile Women with Adenomyosis (n_2_ = 35)	OR [95% CI]	*p*-Value	p_FDR_ ^(a)^	Adjusted OR ^(b)^ [95% CI]	Adjusted *p*-Value	p_FDR_ ^(a)^
VEGF –936*C/T*				0.8255	0.9287		0.8215	0.9242
CC	33 (66.0)	21 (60.0)	Reference			Reference		
CT	14 (28.0)	12 (34.3)	1.35 [0.52, 3.47]			1.35 [0.52, 3.50]		
TT	3 (6.0)	2 (5.7)	1.05 [0.16, 6.80]			1.06 [0.16, 6.98]		
Dominant (CC vs. CT + TT)	17 (34.0)	14 (40.0)	1.29 [0.53, 3.16]	0.5723	0.7358	1.30 [0.53, 3.21]	0.5640	0.7251
Recessive (CC + CT vs. TT)	3 (6.0)	2 (5.7)	0.95 [0.15, 6.0]	0.9560	0.9560	0.96 [0.15, 6.09]	0.9616	0.9616
P_HWE_	0.3797	1.000						
VEGF –634*C/G*				0.3643	0.6886		0.3642	0.6907
CC	32 (64.0)	25 (71.4)	Reference			Reference		
CG	16 (32.0)	7 (20.0)	0.56 [0.20, 1.57]			0.56 [0.20, 1.57]		
GG	2 (4.0)	3 (8.6)	1.92 [0.30, 12.38]			1.92 [0.30, 12.40]		
Dominant (CC vs. CG + GG)	18 (36.0)	10 (28.6)	0.71 [0.28, 1.81]	0.4714	0.7071	0.71 [0.28, 1.82]	0.4737	0.7106
Recessive (CC + CG vs. GG)	2 (4.0)	3 (8.6)	2.25 [0.36, 14.23]	0.3827	0.6889	2.25 [0.36, 14.22]	0.3837	0.6907
p_HWE_	1.000	0.0656						
VEGF –2578*C/A*				0.0116 *	0.0522		0.0106 *	0.0477 *
CC	25 (50.0)	7 (20.0)	Reference			Reference		
CA	17 (34.0)	16 (45.7)	3.36 [1.14, 9.91]			3.47 [1.16, 10.37]		
AA	8 (16.0)	12 (34.3)	5.36 [1.57, 18.25]			5.49 [1.60, 18.85]		
Dominant (CC vs. CA + AA)	25 (50.0)	28 (80.0)	4.00 [1.48, 10.84]	0.0041 *	0.0369 *	4.13 [1.50, 11.32]	0.0037 *	0.0333 *
Recessive (CC + CA vs. AA)	8 (16.0)	12 (34.3)	2.74 [0.98, 7.66]	0.0519	0.1557	2.74 [0.98, 7.68]	0.0516	0.1548
P_HWE_	0.1126	0.7340						

Data are expressed as absolute frequency (number of cases) and relative frequency (%); OR = unadjusted odds ratio; 95% CI = 95% confidence interval; ^(a)^ False Discovery Rate-corrected *p*-value for multiple hypotheses testing; ^(b)^ adjusted by age; HWE = Hardy–Weinberg equilibrium; * significant result: *p*-value < 0.05.

**Table 4:** Three-locus and two-locus haplotype results for association of VEGF gene polymorphisms with adenomyosis risk.

Haplotypes	HF in Women Without Adenomyosis	HF in Women with Adenomyosis	OR ^(a)^ [95% CI]	*p*-Value	Adjusted OR ^(b)^ [95% CI]	Adjusted *p*-Value
Three-locus haplotypes (VEGF *─936/─634/─2578*)						
C–C–C	0.4438	0.2764	Reference haplotype		Reference haplotype	
C–C–A	0.2398	0.3953	2.23 [1.02, 4.90]	0.0457 *	0.26 [1.01, 5.04]	0.0460 *
C–G–A	Na	0.0751	Na	Na	Na	Na
C–G–C	0.1164	0.0247	Na	Na	Na	Na
T–C–A	0.0400	0.0699	1.98 [0.34, 11.57]	0.4496	2.09 [0.32, 13.44]	0.4389
T–C–C	0.0764	0.0726	1.32 [0.31, 5.52]	0.7050	1.33 [0.32, 5.60]	0.6947
T–G–A	0.0502	0.0311	0.87 [0.08, 9.24]	0.9091	0.83 [0.07, 9.77]	0.8821
T–G–C	0.0334	0.0549	1.32 [0.12, 14.63]	0.8236	0.99 [0.85, 1.14]	0.7898
Two-locus haplotypes						
VEGF *─936/─634* ^(1)^						
C–C	0.6820	0.6754	Reference haplotype		Reference haplotype	
C–G	0.1180	0.0960	0.81 [0.26, 2.47]	0.7085	0.81 [0.26, 2.52]	0.7202
T–C	0.1180	0.1389	1.16 [0.42, 3.23]	0.7709	1.18 [0.41, 3.34]	0.7601
T–G	0.0820	0.0897	1.13 [0.33, 3.95]	0.8439	1.13 [0.32, 3.96]	0.8465
VEGF *─634/─2578* ^(2)^						
C–C	0.3415	0.6581	Reference haplotype		Reference haplotype	
C–A	0.5340	0.3504	2.05 [1.03, 4.07]	0.0403 *	2.08 [1.04, 4.16]	0.0396 *
G–A	0.0640	0.1076	3.50 [0.71, 17.09]	0.1222	3.46 [0.71, 16.84]	0.1239
G–C	0.1360	0.0782	0.58 [0.13, 2.48]	0.4594	0.59 [0.14, 2.58]	0.4842
VEGF *─936/─2578* ^(3)^						
C–C	0.5590	0.3097	Reference haplotype		Reference haplotype	
C–A	0.2410	0.4618	2.79 [1.30, 6.01]	0.0086 *	2.86 [1.31, 6.26]	0.0084 *
T–A	0.0890	0.1097	1.99 [0.60, 6.57]	0.2573	2.03 [0.61, 6.77]	0.2506
T–C	0.1110	0.1189	1.56 [0.53, 4.63]	0.4213	1.63 [0.54, 4.95]	0.3882

Note. Data are expressed as relative frequency; alleles in haplotype are described in order of studied gene polymorphisms (VEGF –*936C/T*, VEGF –*634C/G*, VEGF –*2578C/A*); global haplotype association *p*-value for additive three-locus haplotype model: 0.0147; ^(1)^ *p*-value = 0.059 for the global model test; ^(2)^ *p*-value = 0.0079 for the global model test; ^(3)^ *p*-value = 0.058 for the global model test; HF = haplotypes frequencies; Na = not available due to absence or low frequency in one of the two groups; ^(a)^ odds ratio of each haplotype estimated from haplotype-based GLM regression without covariates; ^(b)^ odds ratios of each haplotype adjusted for age (years); 95% CI: 95% confidence interval; * significant result: *p* < 0.05.

**Table 5:** Genotypes frequencies of the studied polymorphisms by adenomyosis severity.

Genetic Models	Infertile Women Without Adenomyosis (*n*_1_ = 50)	Infertile Women with Mild/Moderate Adenomyosis (*n*_2_ = 25)	Infertile Women with Severe Adenomyosis (*n*_3_ = 10)	*p*-Value
VEGF –936*C/T*				0.3360
CC	33 (66.0)	17 (68.0)	4 (40.0)	
CT	14 (28.0)	6 (24.0)	6 (60.0)	
TT	3 (6.0)	2 (8.0)	0 (0.0)	
Dominant (CC vs. CT + TT)	17 (34.0)	8 (32.0)	6 (60.0)	0.2798
VEGF –634*C/G*				0.4446
CC	32 (64.0)	19 (76.0)	6 (60.0)	
CG	16 (32.0)	4 (16.0)	3 (30.0)	
GG	2 (4.0)	2 (8.0)	1 (10.0)	
Dominant (CC vs. CG + GG)	18 (36.0)	16 (24.0)	4 (40.0)	0.5616
VEGF –2578*C/A*				0.0015 *
CC	25 (50.0)	7 (28.0)	0 (0.0)	
CA	17 (34.0)	13 (52.0)	3 (30.0)	
AA	8 (16.0)	5 (20.0)	7 (70.0)	
Dominant (CC vs. CA + AA)	25 (50.0)	18 (72.0)	10 (100.0)	0.0029 *

Data are expressed as absolute frequency (number of cases) and relative frequency (%); * significant result: *p*-value < 0.05.

**Table 6:** Associations between the studied genetic polymorphisms and the odds of dysmenorrhea.

Genetic Dominant Models	Infertile Women Without Clinically Relevant Dysmenorrhea (n_1_ = 66)	Infertile Women with Clinically Relevant Dysmenorrhea (n_2_ = 19)	OR [95% CI]	*p*-Value	p_FDR_ ^(a)^	Adjusted OR ^(b)^ [95% CI]	Adjusted *p*-Value ^(b)^	p _FDR_ ^(a)^
VEGF –936*C/T*								
CC	41 (62.1)	13 (68.4)	Reference			Reference		
CT + TT	25 (37.9)	6 (31.6)	0.76 [0.26, 2.25]	0.6125	0.6125	0.75 [0.25, 2.24]	0.6028	0.6028
P_HWE_	0.2917	1.0000						
VEGF –634*C/G*								
CC	43 (65.2)	14 (73.7)	Reference			Reference		
CG + GG	23 (34.8)	5 (26.3)	0.67 [0.21, 2.09]	0.4794	0.6125	0.66 [0.21, 2.08]	0.4725	0.6028
p_HWE_	0.4429	0.3710						
VEGF –2578*C/A*								
CC	19 (28.8)	13 (68.4)	Reference			Reference		
CA + AA	47 (71.2)	6 (31.6)	0.19 [0.06, 0.56]	0.0019 *	0.0057 *	0.18 [0.06, 0.55]	0.0017 *	0.0051 *
P_HWE_	0.3312	0.1427						

Data are expressed as absolute frequency (number of cases) and relative frequency (%); OR = unadjusted odds ratio; 95% CI = 95% confidence interval; ^(a)^ False Discovery Rate-corrected *p*-value for multiple hypotheses testing; ^(b)^ adjusted by age; HWE = Hardy–Weinberg equilibrium; * significant result: *p*-value < 0.05.

**Table 7:** Associations between the studied genetic polymorphisms and the odds of severe menorrhagia.

Genetic Dominant Models	Infertile Women Without Severe Menorrhagia (n_1_ = 75)	Infertile Women with Severe Menorrhagia (n_2_ = 10)	OR [95% CI]	*p*-Value	p _FDR_ ^(a)^	Adjusted OR ^(b)^ [95% CI]	Adjusted *p*-Value	p _FDR_ ^(a)^
VEGF –936*C/T*								
CC	47 (62.7)	7 (70.0)	Reference			Reference		
CT + TT	28 (37.3)	3 (30.0)	0.72 [0.17, 3.01]	0.6467	0.6467	0.70 [0.17, 2.94]	0.6188	0.6188
P_HWE_	0.3268	1.000						
VEGF –634*C/G*								
CC	48 (64.0)	9 (90.0)	Reference			Reference		
CG + GG	27 (36.0)	1 (10.0)	0.20 [0.02, 1.64]	0.0725	0.1088	0.19 [0.02, 1.60]	0.0672	0.1008
p_HWE_	0.4996	0.0526						
VEGF –2578*C/A*								
CC	25 (33.3)	7 (70.0)	Reference			Reference		
CA + AA	50 (66.7)	3 (30.0)	0.21 [0.05, 0.90]	0.0269 *	0.0807	0.20 [0.05, 0.86]	0.0230 *	0.0690
P_HWE_	0.1042	1.000						

Data are expressed as absolute frequency (number of cases) and relative frequency (%); OR = unadjusted odds ratio; 95% CI = 95% confidence interval; ^(a)^ False Discovery Rate-corrected *p*-value for multiple hypotheses testing; ^(b)^ adjusted by age; HWE = Hardy–Weinberg equilibrium; * significant result: *p*-value < 0.05.

## Data Availability

The raw data involved in this study can be obtained upon reasonable request addressed to Lucia M. Procopciuc (lprocopciuc@umfcluj.ro).

## Abbreviations

IQR	interquartile range
IVF	in vitro fertilization
HMB	heavy menstrual bleeding
HWE	Hardy–Weinberg Equilibrium
OR	odds ratio
Q-Q plots	quantile–quantile plots
MUSA	morphological uterus sonographic assessment consensus
PBAC	pictorial blood loss assessment chart
PCR	polymerase chain reaction
RFLP	restriction fragment length polymorphism
VEGF	vascular endothelial growth factor
VSA	visual analog scale
3′-UTR	3′ untranslated region
5′-UTR	5′ untranslated region
95% CI	95% confidence interval
